# Occupational hygiene risk assessment at light speed—a study for protecting worker health and safety in the biopharmaceutical industry

**DOI:** 10.3389/fpubh.2025.1559588

**Published:** 2025-06-25

**Authors:** Paul Kayser, Leonid Turczynowicz, Sharyn Gaskin

**Affiliations:** ^1^Pfizer Kalamazoo, Occupational Safety & Industrial Hygiene, Pfizer Inc, Kalamazoo, MI, United States; ^2^Adelaide Exposure Science and Health Laboratory, School of Public Health, University of Adelaide, Adelaide, SA, Australia

**Keywords:** occupational health, industrial hygiene, biopharmaceutical, pharmaceutical exposure assessment model, hazardous substance

## Abstract

**Background:**

Hazardous substances are ubiquitous in the workplace and improperly controlled exposure may result in severe illness and death. Occupational exposure models can be used to predict the level of exposure workers may experience while performing tasks and thus determine “acceptability” or compliance against an applicable exposure standard. This reflects a prospective assessment approach providing useful information and critical to the biopharmaceutical industry where a high degree of novel exposure scenarios are present.

**Research aim:**

This research sought to provide practical insights and recommendations of suitable occupational exposure models for use in the biopharmaceutical industry to support a new, light speed’ pace of biopharmaceutical process development, scale-up and manufacturing. This was achieved through the identification and critical review of the most recent and innovative occupational exposure models to assess their suitability for supporting these novel industry initiatives while also informing future research opportunities.

**Methods:**

A systematic literature search strategy was developed and conducted. Studies that met the inclusion criteria were identified for further review of potential exposure models. Models were screened at a high level of detail for inclusion in a critical review of components, including their mechanisms, capabilities, level of validation and “acceptability” for use.

**Inclusion criteria:**

Models were selected for critical review on the basis of their availability as an electronic tool, endorsement by an appropriate advisory body based on field validation and suitability for exposure assessment of inhalation hazards relevant to the biopharmaceutical manufacturing and process development industry.

**Results and conclusions:**

The basis behind key elements such as control banding, heuristic structure, multiplying factors, mass balance and multiplying factor-mass balance hybrid tools were reviewed and seven tools were critically assessed for suitability. ART was recommended as the most appropriate tool for use by industrial hygiene professionals; STOFFENMANAGER® was recommended for use by safety professionals with chemical safety experience; and the COSHH e-tool was recommended as a useful tool for process engineers, operations managers, and operators.

## 1 Introduction

Millions of workers are reportedly exposed to hazardous substances through their occupation each year (1). Diseases associated with exposure to hazardous substances in the workplace are estimated to cause 2.3 million global deaths per year and an estimated economic cost between 1.8 to 6 percent of country-specific gross domestic product (2). Understanding exposures requires either measurement or modeling tools. There has been a growing focus toward models and model development and in areas such as the biopharmaceutical industry there are existing gaps in chemical exposure assessment lends itself to a focus toward the use of suitable models.

Over the past 35 years the biopharmaceutical industry has made a major contribution to the extension and improvement in quality of human life through the development of many vaccines and related therapeutic agents. This industry employs some, 5.5 million workers (3), many of whom may have the potential for exposure to hazardous substances through associated industry manufacturing processes.

Medicines contain active pharmaceutical ingredients (APIs) (4) and are synthesized through either organic chemical synthesis or biological expression (5, 6). Biopharmaceutical workers have the potential for much higher occupational exposure to APIs than patients (7), with immediate, acute or chronic exposures leading to mild to extremely severe health outcomes. For example, regular exposure to Minocycline has the potential to cause a permanent blue-grey discoloration of the sclera, fingernails, teeth and skin (8) while any unprotected exposure to highly hazardous oncolytic medicines may have carcinogenic, genotoxic, teratogenic, reproductive or specific organ toxicity effects (9).

Acute exposures to APIs in the opioid and anesthesia family have the potential to cause symptoms such as drowsiness and/or confusion (10), substantially increasing the likelihood of workplace accidents and injury (7).

Chronic exposures may also result in sensitization or the development of tolerance, removing medicine as an option for future disease treatment (7) as reported by Farshad et al. where significantly higher penicillin resistance in *Streptococcus pneumoniae* strains was observed in isolates from pharmaceutical workers in Tehran (11). Similar outcomes have been observed in studies of pharmaceutical workers in Bangladesh (12) and Ireland (13). Increased work history and employment as process operators and maintenance technicians were recognized as influencing factors.

These examples reinforce the significance of understanding exposures and implementing risk mitigation, particularly as this industry sector generates novel substances of specific toxicities and novel manufacturing methods on a regular basis.

### 1.1 Basic principles of protecting pharmaceutical workers from exposure to hazardous substances

The cornerstone of occupational health and hygiene is occupational risk assessment comprising hazard identification, substance characterization in terms of its toxicology, exposure assessment, risk characterization and the subsequent implementation of effective risk controls to eliminate or minimize exposure. This also includes confirmatory monitoring to ensure controls remain effective (14). Various models and guidelines have been developed by organizations and countries to assess occupational health risk in the workplace. Each model has its own unique principles, with advantages and disadvantages. However, the differences in methodologies, and comparisons of effectiveness of these models in different industries have not been extensively reported (2).

### 1.2 Identifying an applicable exposure profile

To assess the risk of a hazardous substance, exposure information is needed to differentiate between a safe (and therefore acceptable) and unsafe (and therefore unacceptable) level of exposure (15). Occupational exposure limits (OELs) are established to define the airborne concentration that healthy workers may be exposed to, 5 days per week, 8 h per day, over a 40-year working life and not experience any significant adverse health effects across the majority of the worker population (7).

There are few published OELs for biopharmaceutical medicines (7, 16), especially novel, early-stage substances where the level of human health data required to derive an OEL does not yet exist. To facilitate early-stage assessments, a system of occupational exposure bands (OEB) was developed (15, 17) (see Table 1).

**Table 1:** Occupational exposure banding system adapted from Graham et al. (17).

Occupational exposure band	Range (μg/m^3^)	Relevant compounds	Example
OEB 1	≥ 1000	Very low toxicity/potency	Caffeine
OEB 2	100–< 1000	Low toxicity/potency	Tetracycline
OEB 3	10–< 100	Intermediate toxicity/potency	Statins
OEB 4	1–< 10	Potent/toxic compounds	Early discovery APIs
OEB 5	0.1–< 1	Highly potent/toxic compounds	Toxic oncology drugs and steroids
OEB 5 Special Case	<0.1	Especially potent/toxic compounds	Especially potent/toxic protein nucleic acids

OEBs are conservatively assigned and correlate with established organizational handling and control practices. As the amount of substance-specific health data, and therefore certainty of the OEB classification increases, the OEB classification and associated controls may be updated (16–18).

Biological pharmaceuticals, including proteins, peptides, antibodies, DNA and RNA, bacterial and viral-based therapeutics, have been argued to have a simplified banding system, based on the typically closed nature of manufacture required to ensure process sterility and lower bioavailability through non-intravenous absorption mechanisms. Graham et al. outlines a rough guide to the application of occupational exposure bands to different types of biopharmaceutical compounds (17), which may be of use to professional practice.

### 1.3 Exposure assessment

Workers that are conducting similar tasks, at similar frequencies using similar handling protocols and operational controls can be grouped together, for the purpose of occupational health risk assessment, into Similar Exposure Groups (SEGs). All members of a SEG are therefore assumed to have a similar exposure profile to the substance of concern, this is important as it allows for inferential analysis and reduces the number of exposure assessments that need to be undertaken (14, 19). The aim of the Occupational Hygienist is to estimate the exposure profile of a given SEG to the agent(s) of concern and determine if it is acceptable (14). This can be achieved in several ways as subsequently described.

#### 1.3.1 Biological monitoring

Biological exposure monitoring uses biomarkers, typically from blood or urine, to determine the amount of hazardous agent absorbed into the body of the worker. Biological monitoring (BM) can be especially useful for assessments of hazards with a dermal exposure pathway and for verification of exposure control efficacy (20) but it requires consideration of substance kinetics; is costly to develop and deliver at scale; can be affected by non-workplace factors, and may present ethical concerns associated with ‘the veracity of informed consent’ and independence of health professionals overseeing the process (21). In addition, individual variability in excretion rates and the need to ensure sampling consistent with known toxicokinetic understanding presents further challenges (22) Most importantly BM is retrospective, as exposure is historical and has already occurred, with the worker having already absorbed enough of the substance of concern to be measurable or to have elicited a biologically detectable change (21, 23).

#### 1.3.2 Quantitative airborne exposure monitoring

Airborne sampling uses a calibrated sampling pump to draw air at a continuous rate through specific collection media over a defined time period. The sample is sent to an accredited laboratory for testing and determination of the amount of substance collected. The mass per air volume sampled then provides a measured air concentration. Airborne sampling occurs either as a static/area sample or as a personal monitoring sample. For personal monitoring, the pump is worn by the worker throughout a representative period of their workday and the air is drawn directly from the worker’s breathing zone. Only personal monitoring can be used to compare to an established OEL and assess worker exposure (24, 25).

While quantitative exposure assessment is considered the ‘gold standard’ for determining worker exposure (26), there are some important limitations. The planning, set up, handling and use of the monitoring equipment is critical to ensuring high quality data are collected. All sampling activities should be conducted by competent, trained personnel and the results, calculations and inferences overseen by a certified industrial/occupational hygienist (27). In addition, a suitable collection medium and a sufficiently sensitive, validated laboratory analysis protocol must be available for the hazardous substance of interest. While usually available for common substances, such as methylene chloride, it will often not be the case for novel biopharmaceutical substances. These substances require collection and analysis methods to be developed, which requires time, cost; and may not always be successful (14, 28, 29). Finally, there may also be limitations on the ability to complete airborne sampling of workers due to the stringent control of activities and materials permitted in higher grade clean rooms often used for the manufacture/fill-finish of sterile medicinal products (30).

#### 1.3.3 Exposure modeling

Biopharmaceutical plants have the opportunity for hundreds, if not thousands of workplace exposure scenarios. An efficient and robust process that facilitates a suitably competent professional to use a model to assess the acceptability of a process, and its exposure controls, against an applicable reference standard (with a conservative degree of confidence) is critical for worker protection and effective resource allocation (31).

Traditionally, organizations relied on certified industrial hygienists to apply their experience and training to make a judgment on process acceptability and the need for air sampling (31, 32). However, current research would suggest the accuracy of such assessments, “based on subjective professional judgment is low, not statistically different from random chance, and tends to underestimate exposures” (31).

Exposure assessment accuracy has been shown to be significantly improved by using standardized checklists, algorithms, and models (31, 32) and the use of exposure modeling has been deemed as “essential in almost all relevant contexts for exposure science” (33). This ‘essentiality’ is, however, constrained by the need to ensure exposure models used reflect reality through suitable and robust validation techniques using reference measurement methods. Through validation, a robust exposure model offers many advantages in the examination of workplace or non-occupational exposures by enabling tools such as sensitivity and variability analysis to be used. The former enables an understanding of the most critical variables that affect exposures while the latter consider the outcomes of variability across influencing parameters. Analysis outcomes thus enable a focus on the acquisition of suitable Australian data across those parameters enabling improvement in model predictions.

While various standardized exposure assessment tools and models have been proposed, the evaluation of these models for specific exposure scenarios is unfortunately scarce, and assessment/validation of tool suitability for specific industries is recommended (31, 34).

### 1.4 Occupational exposure modeling in the biopharmaceutical industry—a case study

The following case study helps demonstrate the need for high quality exposure assessment modeling tools that can be applied across the biopharmaceutical process development and production pipeline.

On the 2nd of December 2021, the partnership of Pfizer-BioNTech received emergency use approval for COMIRNATY®, the mRNA vaccine against SARS-CoV-2. This approval represented a speed of therapeutic development, manufacturing scaleup and commercial launch that was unprecedented (35, 36) in an industry where the typical time from early phase research and development (R&D) to market approval takes up to 15 years (37). This drastic increase in the speed of development, combined with the novel modalities and ‘increasing complexity and potency’ of the newest wave of biotherapeutic medicines, raised questions as to whether current occupational hygiene approaches, such as those used to manage the risks arising from small molecule medicinal manufacture, are still suitable for today’s modern biopharmaceutical research and manufacturing environment (17).

## 2 Research aims

The aim of this research was to:

- Conduct a systematized literature review of the current approaches and models used for occupational hygiene exposure assessment.
- Conduct a critical analysis of identified occupational hygiene exposure models against the needs of the growing pace of the biopharmaceutical industry.
- Propose a current model, or models, best placed to meet the needs of occupational hygiene exposure assessment in the biopharmaceutical industry.
- Reflect on opportunities for future research on occupational exposure assessment modeling that may be useful to the biopharmaceutical industry.

## 3 Methods

### 3.1 Literature search, refinement, screening, and eligibility assessment

A systematized approach (38) was used to provide a summary of the most impactful, innovative, and recent research on the occupational health exposure models that have the potential for application to biopharmaceutical process development, scale up and manufacturing. A key word search strategy was developed and applied to the PubMed, Web of Science and Scopus literature databases (see Supplementary Tables S1–S3). Results were further supplemented with outputs from grey literature searches using Google Scholar and applicable websites from relevant institutions and regulatory organizations. The combined output was refined through the following criteria—English publications, publication date in the last ten years with topics associated with public health, and environmental and occupational health and safety.

Endnote (15) and Reference Manager (39) bibliographic software packages were used for reference management including duplicate identification and removal, with supplementation through manual review. Resulting records were exported to the online Rayyan application for system reviews (40), with titles and abstract screened against a predetermined inclusion criterion, being describing the use of methodologies for occupational exposure assessment or modeling that is not based on biomarker or air sampling data analysis. Publications were excluded where they did not meet this criterion or where the study populations were not from an industrial occupational setting.

**Figure 1:**
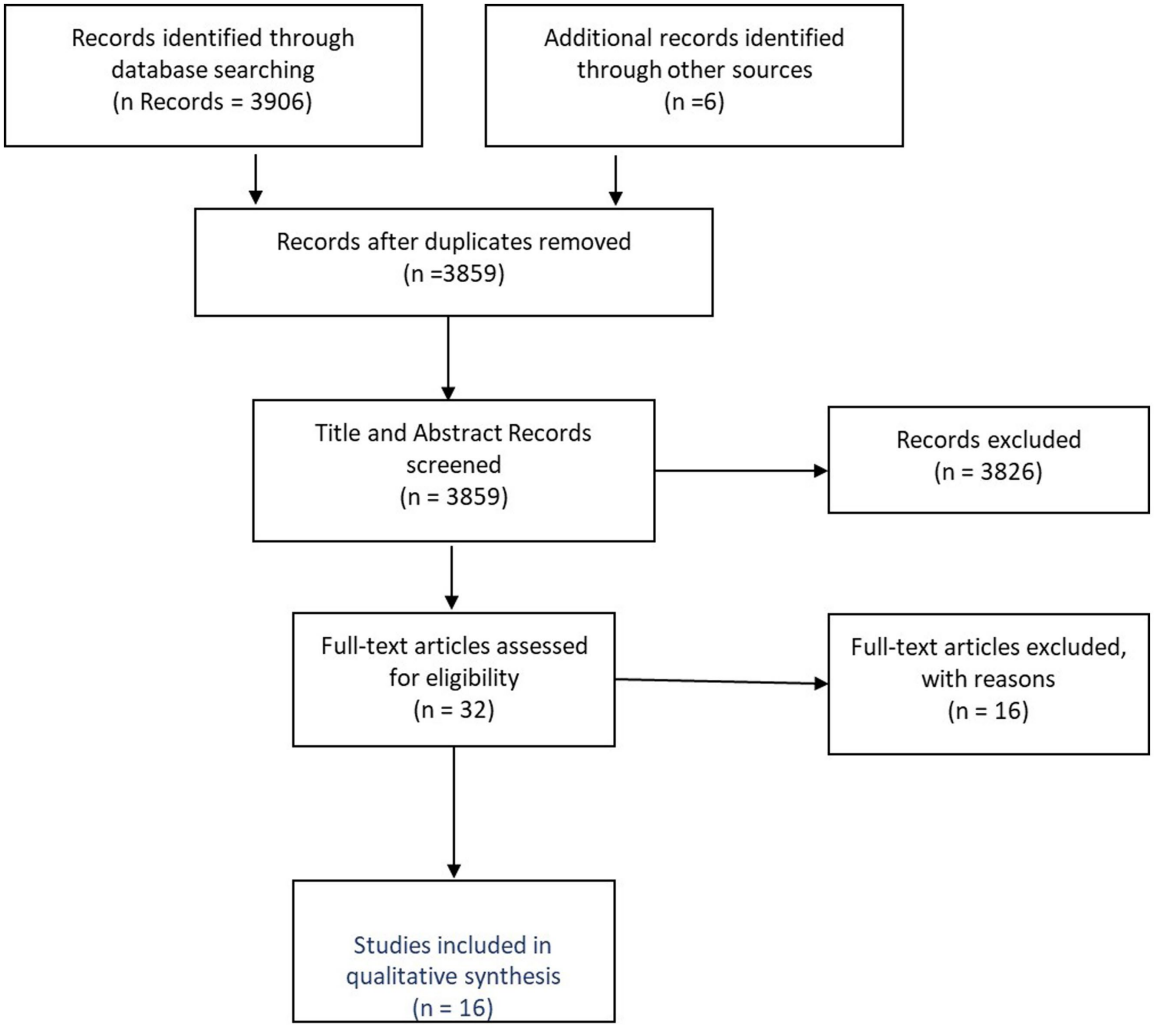
Prisma diagram reflecting literature search and screen output.

Full text screening confirmed relevance against the inclusion criteria (see Supplementary Tables S4, S5) with sixteen publications identified for critical review and data extraction (41). The Prisma Diagram (Figure 1) reflects the literature search and screen output.

### 3.2 Assessment criteria for inclusion into detailed critical review

Models were selected for detailed critical review on the basis that they were identified as:

- Suitable for assessment of inhalable hazardous materials relevant to biopharmaceutical manufacturing, and
- Available as an electronic tool (web-based application, downloadable application), or controlled spreadsheet (thereby eliminating the potential for user-based calculation error), and
- Endorsed by a suitable regulatory or advisory body.

or

- An important type of recently published exposure model that was not otherwise represented (and subject to regulatory review).

or

- Identified as providing a useful tool (subject to further regulatory evaluation), relevant to occupational exposure control that would be of interest to the biopharmaceutical community.

### 3.3 Occupational exposure assessment methodology evaluation

Each study was reviewed in detail and the following information was extracted, as available.

- Assessment name.
- Earliest publication reference.
- Model tier.
- Brief model description.
- Modeling capabilities.
- Tool type.
- Summary of noted model advantages, disadvantages, or concerns.
- Web address for tool access, source references and
- Critical review inclusion decision with rationale.

Results were tabulated and listed in alphabetical order by model name (Table 2).

**Table 2:** Results of occupational exposure model information review.

Methodology name	Earliest identified publication references	Model Tier	Brief method description	Endorsing bodies	Modeling capabilities	Tool type	Summary of noted advantages of model/tool	Summary of noted cautionary concerns or disadvantages of model	Available from	Table data references	Included into detailed synthesis	Rationale for acceptance
Advanced Reach Tool (ART)	Fransman et al. (2011) (56)	Tier 2	Task based mechanistic model, applies “multiplying factors” to a near field-far field framework	European Chemical Agency (ECHA)	Inhalable dust Vapors Mists	Web based	Only Tier 2 model endorsed by ECHA Openly published model Capable of calculating combined daily exposure from multiple activities Allows Bayesian modeling with incorporation of real-world exposure data Provides quantitative exposure estimates with uncertainty analysis Calibrated with over three thousand exposure measurements More accurate/precise than TRA and Stoffenmanager®	Requires high level of expertise to use appropriately Not currently capable of modeling gases, fibers, and fume Can underestimate low exposures and overestimate high exposures Appropriateness of estimating quantitative results with confidence intervals from semi-qualitative data questioned Correctness of linear moisture effect on dustiness questioned	https://www.advancedreachtool.com/	Hofstetter et al. (2013) (42), Bekker et al. (2016) (52), Sailabaht (2018) (45), Shandilya et al. (2019) (54), Cherrie et al. (2020) (26), Koivisto et al. (2021) (50), and Schluter et al. (2022) (44)	Yes	Endorsed Tier 2 model
ANSES agency standard CB framework (Anses Tool)	Ostiguy C et al. (2010) (57)	NA	Flowchart methodology to apply control bands to nano materials	None identified	NA	Paper based	Provides a control banding framework for the hazard classification of nano materials	Does not consider process characteristics, only physical properties of the source Highly conservative model	Microsoft Word—Control banding UK version finale.docx (anses.fr)	Gridelet et al. (2015) (18)	No	Not an exposure model
Control banding method for handling of powders and nanomaterials	Gridelet et al. (2015) (18)	Tier 1	Multiplying factors exposure risk assessment tool	None identified	Powders and solid aerosols including nano materials	Paper based	Simple model Can be used for lab to industrial scale Considers both the substance and process properties	No evidence of validation identified No uncertainty calculation included with quantitative output	https://www.ncbi.nlm.nih.gov/pmc/articles/PMC4331195/	Gridelet et al. (2015) (18) and Gul et al. (2017) (58)	Yes	Representative of a simple Tier 1 model
CB Nanotool	Paik et al. (2008) (59)	Tier 1	Multiplying factors exposure risk assessment tool	None identified	Powders and solid aerosols including nano materials	Spreadsheet	Easy to use with simple additive parameters Provides risk score applied to a risk matrix	Focus is nano and limited to 100mg scale	Lawrence Livermore National Laboratory (llnl.gov)	Gridelet et al. (2015) (18)	No	Very small scale
Computerized Inherent occupational health index (Computerized IOHI)	Pandian et al. (2013) (60)	Not identified	Occupational health assessment of R&D phase processes	Not identified	Not identified	Downloadable application	R&D stage improved usability Limits manual calculation errors	Not applicable to all stages of biopharmaceutical development	Not identified	Alhamdani et al. (2018) (46)	No	Not an electronic tool Not applicable to all stages of biopharmaceutical development
Control of Substances Hazardous to Health Regulations—COSHH-Essentials e-tool	Garrod et al. (2007) (61)	NA	Simple exposure risk assessment	UK Health and Safety Executive (HSE)	Solids and liquids	Web based	Simple tool Allows basic assessment using H-phrases, vapor pressure or simple qualitative dustiness measure and frequency Provides fact sheets recommending generic control strategies based on assessed risk level	Not regarded by the HSE as a formal modeling tool	Getting started—COSHH e-tool (hse.gov.uk)	Cherrie et al. (2020) (26)	Yes	Example of one of the earliest published tools. Provides useful information to non-safety professionals or those with minimal background in chemical exposure risks and controls
Decision tree-based method	Groso et al. (2016) (55)	NA	Flow chart/Decision tree	None identified	Small scale nano materials	Paper based	Addresses use of nano materials in research laboratory environment	Difficult to adapt to an industrial environment	Engineered nanomaterials: toward effective safety management in research laboratories—PMC (nih.gov)	Gridelet et al. (2015) (18)	No	Not an exposure model
Dow Chemical Exposure Index	Marshall et al. (1995) (62)	Not identified	Not known	None identified	Not identified	Not known	Earliest identified model. Assess health impact from chemical release incidents (acute exposure only)	Model focus is public health rather than occupational health risk. Model does not consider chronic exposure	Not known	Alhamdani et al. (2018) (46)	No	Not an occupational exposure model
Environmental health and safety (EHS) method	Koller et al. (2000) (63)	Not known	Not known	None identified	Not identified	Not known	Assessment of safety and environmental risk including health effects	Does not consider the toxic exposure risk	Not known	Alhamdani et al., 2018 (46)	No	Model does not fully assess occupational health exposure risks
Estimation and Assessment of Substance Exposure (EASE) tool	Tickner et al. (2005) (64)	Not identified	Not known	UK Health and Safety Executive (HSE)	Not identified	Not known	Not identified	Crude and unreliable	Not identified	Cherrie et al. (2020) (26)	No	More “appropriate” models identified
Extended Heuristic framework	Ng and Hassim (2015) (65)	Not identified	Guideline and Heuristic framework including “Inherently safer design (ISD)” principles	Not identified	Not identified	Paper based	Build on Heuristic framework to incorporate ISD principles Combined Simplifies heuristic framework & includes concepts of inherently safer design	Not identified	Not identified	Alhamdani et al. (2018) (46)	No	Not an electronic tool
Einfaches Maßnahmenkonzept für Gefahrstoffe tool—EMKG-Expo-Tool	ECHA (2016) (66)	Tier 1	Task based quantitative exposure assessment of inhalable dusts and liquids	European Chemical Agency (ECHA) German Federal Institute for Occupational Safety and Health	Dust and liquids	Downloadable application	Simplified quantitative exposure assessment. Recommended for REACH assessment	Not suitable for assessment of dusts generated by abrasive processes, fumes, gases, spraying, pesticides, wood dusts or substances that are carcinogenic, mutagenic or poses reproductive toxicity	https://baua.de/EN/Topics/Chemicals-biological-agents/Hazardous-substances/REACH-assessment-unit/EMKG-Expo-Tool.html	Cherrie et al. (2020) (26), Schlüter et al. (2022) (57), and BAUA website (67)	No	Specific model not a subject of focus for any papers reviewed as part of this synthesis
Graphical Inherent occupational health index (Graphical IOHI)	Hassim et al. (2013) (68)	Not identified	Occupational health assessment of R&D phase processes	Not identified	Not identified	Graphical paper based	R&D stage, graphical tool for improved usability	Not identified	Not identified	Alhamdani et al. (2018) (46)	No	Not an electronic tool
Health Quotient Index (HQI)	Hassim and Hurme (2010) (68)	Not identified	Occupational health assessment of early process development (preliminary design phase)	Not identified	Not identified	Paper based	For preliminary design phase	Potential for mistakes through manual calculations	Not identified	Alhamdani et al. (2018) (46)	No	Not a computer-based tool
Heuristic framework	Ng et al. (2014) (69)	Not identified	Guideline and Heuristic framework	Not identified	Not identified	Paper based	Incorporated IOHI, HQI and OHI tools with guidelines for selection based on known process parameters	Not identified	Not identified	Alhamdani et al. (2018) (46)	No	Not a computer-based tool
Hybrid approach for fugitive emissions estimation	Ng et al. (2017) (70)	Not identified	Estimation of fugitive emissions for process development and design stages	Not identified	Not identified	Not identified	Estimation of fugitive emissions for process development and design stages	Does not cover full range of pharmaceutical development	Not identified	Alhamdani et al. (2018) (46)	No	Specific model not a subject of focus for any papers reviewed as part of this synthesis
Industrial Hygiene Exposure Scenario Tool (IHEST)	Arnold et al. (2015) (32)	NA	Information and Collation tool	American Industrial Hygiene Association (AIHA)	Not a model Basic characterization	Spreadsheet	Supports industrial hygiene professionals by providing a consistent method for data collection	Not in itself an exposure modeling tool	https://www.aiha.org/public-resources/consumer-resources/apps-and-tools-resource-center/aiha-risk-assessment-tools/ih-oehs-exposure-scenario-tool-ihest	Arnold et al. (2016) (32)	Yes	Useful tool for preliminary data collection
Inherent benign-ness indicator (IBI)	Srinivasan and Nhan (2008) (71)	NA	Assess Safety and Environmental aspects of processes	Not identified	Not identified	Not identified	Assess Safety and Environmental aspects of processes	Occupational health risk is not the main focus of the assessment	Not identified	Alhamdani et al. (2018) (46)	No	Occupational health risk is not the main focus of the assessment
Inherent occupational health index (IOHI)	Hassim and Hurme (2010) (72)	Not identified	Occupational health assessment of R&D phase processes	None identified	None identified	Paper based	R&D stage	Potential for mistakes through manual calculations	Not identified	Alhamdani et al. (2018) (46)	No	Not an electronic tool Not applicable to all stages of biopharmaceutical development
Inherent safety, health, and environmental evaluation tool (INSET) Toolkit	INSIDE Project (2001) (73)	Not identified	Assessment of safety and environmental risk	Not identified	Not identified	Not identified	Model uses leak rates to estimate emissions and combines with health hazards identified from R-phrases	Complex model, requiring detailed process information. May not be available for all phases of development.	Not identified	Alhamdani et al. (2018) (46)	No	R-phrases is obsolete
MEASE 2	ECHA (2016) (66)	Tier 1	Process based occupational exposure model	European Chemical Agency (ECHA)	Metals and inorganic solids and liquids	Not identified	Suitable for exposure risk assessment to metals and inorganic substances Recommended as suitable for REACH assessment	Not suitable for assessment of organic substances	https://www.ebrc.de/tools/downloads.php	ECHA (2016) (66) and Schlüter et al. (2022) (44)	No	Model limited to inorganic substances
Near-field, far-field (NF-FF) deterministic model	Spencer (2007) (74)	Not identified	Mass Balance based model	Not identified	inhalation exposure to solvents	Not identified	Reported as reliable for inhalation exposure to solvents	Not identified	Not identified	Hofstetter et al. (2013) (42)	No	Limited use case in biopharma
Occupational health index (OHI)	Hassim and Hurme (2010) (75)	Not identified	Occupational health assessment of late process development (engineering phase)	Not identified	Not identified	Paper based	For engineering design phase	Potential for mistakes through manual calculations	Not identified	Alhamdani et al. (2018) (46)	No	Not an electronic tool Not applicable to all stages of biopharmaceutical development
Occupational health hazard index (OHHI)	Johnson (2001) (76)	Not identified	Allow comparison assessment and ranking of alternate chemical synthesis pathways	Not identified	Not identified	Not identified	Occupational health focused assessment of alternate chemical synthesis pathways	Impractical, require detailed process information. Assessment of fugitive emissions inefficient.	Not identified	Alhamdani et al. (2018) (46)	No	Not applicable to all stages of biopharmaceutical development
Precautionary Matrix for Synthetic Nanomaterials	Höck et al. (2010) (77)	NA	Risk guidance for users of nanomaterials	Not identified	NA	Guide	Provides information on potential occupational exposure risks associated with the use of nano materials during production, handling, and waste disposal	Not a risk assessment method as such	Not identified	Gridelete et al. (2015) (18)	No	Not an exposure model
Process Route Healthiness Index (PRHI)	Hassim and Edwards (2006) (78)	Not identified	Allow comparison assessment and ranking of alternate chemical synthesis pathways	Not identified	Not identified	Not identified	More advanced than OHHI	Very complex, requiring detailed process information	Not identified	Alhamdani et al. (2018) (46)	No	Not applicable to all stages of biopharmaceutical development
Source Path Receptor (SPR) Approach	Alhamdani et al. (2018) (46)	Not identified	Environmental risk assessment	Not identified	Environmental risk	Not identified	Environmental risk assessment of fugitive emissions	Not intended for occupational exposure health risk assessment	Not identified	Alhamdani et al. (2018) (46)	No	Occupational health risk is not the main focus of the assessment
Source Path Receptor-Layers of Protection SPR-LOP hybrid tool	Alhamdani et al. (2018) (46)	Not identified	Accesses health risk via assessment of fugitive emissions and the layers of protection that may prevent exposure	Not identified	Process risk from very simple to highly complex	Paper based	Very comprehensive, considers lots of factors as part of the SPR pathway and layers of protection including strength of the e management system and culture	Very complex and requires a high degree of expertise and time to complete. Output is a relative health risk score not a predicted exposure. No uncertainty measures provided for output	Not identified	Alhamdani et al. (2018) (46)	Yes	A good example of a mass-balance-based model
Stoffenmanager	Marquart et al. (2008) (79)	Tier 1.5 or 1/2 hybrid	a web-based control banding tool and occupational exposure assessment modeling tool	European Chemical Agency (ECHA)	Control banding Quantitative exposure of vapors, low volatility liquid aerosols, and dust. Inhalation and dermal exposure	Web based	More advanced than most exposure modeling tools. Conducts both airborne and dermal assessments Can combine multiple tasks to generate a TWA Validated with over seven thousand measurements	Potential to underestimate low exposures and overestimate high exposures. Calibration data is not publicly available Concerns raised about legitimacy of model validation/calibration High level of training and support recommended to use tool effectively	https://app.stoffenmanager.com/	Bekker et al. (2016) (52), Cherrie et al. (2020) (26), ECHA (2016) (66), Koivistio (2021) (50), Schlüte et al. (2022) (44), and Spinazzè et al. (2019) (34)	Yes	Considered the most “robust” REACH recommended exposure model
Structured deterministic model (SDM 2.0)	Arnold et al. (2015) (32)	Tier 1	Simple heuristic tool for inhalation exposure assessment	AIHA	vapors, aerosols, fibers, and particulates	Spreadsheet	Simple model requiring minimal inputs. Includes detailed explanations and user information. Easily identify clear overexposure and likely non-detect scenarios	Better for “pure volatile and semi-volatile” substances Does not include parameters for dustiness	https://license.umn.edu/product/structured-deterministic-model-sdm-20	Huizen (2023) (47) and Arnold et al. (2016) (31)	Yes	A good example of a simple to use basic exposure model with relatively wide application
Substance, reactivity, equipment, and safety technology (SREST)	Shah et al. (2003) (80)	Not identified	Safety and environmental aspects assessment	Not identified	NA	Not identified	Assessment of safety and environmental risk	Occupational health risk is not the main focus of the assessment. Limited health risk assessment capability does not cover toxic exposure risk.	Not identified	Alhamdani et al. (2018) (46)	No	Occupational health risk is not the main focus of the assessment
Targeted Risk Assessment (ECETOC TRA) tool	Not identified	Tier 1	Modifying factors based preliminary process exposure estimation modeling tool	European Chemical Agency (ECHA)	Inhalation and dermal exposure to dust and volatile liquids	Not identified	Conservative preliminary estimation suitable for a wide variety of use scenarios. Unlike other models TRA uses HSE exposure data originally incorporated into the EASE model rather than the chemical and physical properties of the substance	Not suitable for assessment of fibers, aerosols and mists, fumes, gases, suspended solids. Caution recommended if used for carcinogenic, mutagenic or substances with reproductive toxicity. Not as widely validated as some other models.	Not identified	Cherrie et al. (2020) (26), ECHA (2016) (66), Hofstetter et al. (2013) (42), and Schlüter et al. (2022) (44)	No	Not suitable for carcinogenic, mutagenic or substances with reproductive toxicity

Models are listed alphabetically by model name.

### 3.4 Critical review methodology

A summary of the key investigation findings most applicable to application in the biopharmaceutical industry was presented based on relevance, as determined by expert elicitation. A subset of the most widely reported models were field tested to facilitate a deeper understanding of their function, data entry requirements, usability, outputs, and potential limitations using two scenarios common in the biopharmaceutical industry, being (1) preparation of a stock solution of a stock hydrochloric acid and (2) charging of a tank with diatomaceous earth.

## 4 Results and discussion

### 4.1 Systematized literature review

Sixteen references were included into the systematized literature review; eight studies presented predictive exposure modeling tools against specific use cases, four studies validated the prediction capabilities of various models, six studies reported on the validity of the theoretical background used in various exposure models and one conference paper presented on pre-air sampling exposure assessment. All identified models have previous published evaluations, so the purpose was not to re-evaluate those but to provide rapid initial feedback on applicability of these models to the case study scenarios.

### 4.2 Model identification and assessment

Thirty-two models were identified through detailed literature review (Table 2); of these three were spreadsheet applications, two were computerized downloadable applications and three were web-based e-tools. Sixteen tools were only discussed at a high level for historical context and full detailed information on the nature of the tools was not reviewed. Seven models met the inclusion criteria and were selected for detailed critical analysis.

### 4.3 Critical analysis

#### 4.3.1 Exposure models: overview

##### 4.3.1.1 Model uses

The European Regional Chapter of the International Society of Exposure Science 2022 expert workshop defined exposure models as having two main uses. These are (1) for Chemical Registration, as required under REACH regulations, where all European manufacturers and importers are required to complete a Chemical Safety Assessment to identify and quantitate acute and chronic dermal and inhalation exposure risk of all anticipated exposure scenarios for registering chemicals and (2) to assist employers in meeting their duty of care as part of a workplace exposure control program (42, 43). In either case, in the absence of representative real-world air sampling data, appropriate models must be used (26, 44). Sailabaht et al. noted that exposure models can be used to describe the relationship between emissions and concentrations, “reconstruct historical exposures” and to assess and manage the risks of potential future exposures (45).

There are two primary types of exposure model. Tier 1 models are for relatively simple assessments, require limited information and provide conservative assessment results (26, 42, 45) while Tier 2 models are more complex, require a higher level of expertise and process knowledge to complete with results exhibiting a higher degree of accuracy (26, 42, 44, 45).

Variability and uncertainty are significant concerns for exposure models. Potential sources of variation include the physical state, temperature, moisture content, density, dustiness, and particle size of the source material, environmental factors such as room temperature, humidity and air flow; process-specific variables such as indoor mixing rates and local exhaust ventilation efficiency; and worker-related factors such as training, competency, experience, supervision and culture (26, 44, 46). Higher tier models will represent this by providing a measure of uncertainty (26). In general terms, the greater the uncertainty, the greater the conservatism in model outputs leading to increased risk estimates and increased costs of risk mitigation. On this basis any reductions in uncertainty (as a result of new data acquisition) aid in improved confidence concurrent with improvements in the cost-efficacy of risk mitigation measures.

#### 4.3.2 Review of select exposure models

Various types of modeling tools were identified including simple checklists; heuristic models; multiplying-factor models; mass-balance-based models and models that incorporate combinations of the above. A summary of key models and model mechanisms is subsequently presented in order of increasing complexity.

##### 4.3.2.1 The structured deterministic model (SDM 2.0)

SDM 2.0 is the checklist approach developed by Arnold et al., incorporated into a simple Excel based tool and recommended by the AIHA to improve the accuracy of exposure assessment by professional judgment (37, eight2) (see Supplementary Table S6 for model summary). Each checklist uses applicable heuristics; rule of ten and ‘Vapor Hazard Ratio’ or Antoines and Raoult’s laws to rate the level of exposure control and health risk to the operator “based on the 95th percentile exposure” (32, 47). Huizen suggests that the tool works best for volatiles at the exposure extremes and that the particulate tool is simplistic, not discriminating by dustiness (47).

##### 4.3.2.2 The control of substances hazardous to health (COSHH) essentials tool

COSHH essentials e-tool is a simple risk assessment created by the UK Health and Safety Executive (HSE) (48) (see Supplementary Table S7 for a summary of the COSHH e-tool). The tool uses a series of risk matrices to generate a summary report (see Supplementary Table S8). The calculations behind the tool have been fully published and the tool extensively tested, typically reported as highly conservative, and overestimating exposure (48, 49).

#### 4.3.3 Modifying factor based models

Modifying factor-based models are empirical models that create a base estimate of exposure. The user-selected factors apply specific risk multipliers, calculating a theoretical final exposure level (26, 44, 50). Gridelet et al. proposed a simple modifying factors approach based on the occupational hazard bands model for powders and nano materials (18) (see Supplementary Table S9 for tool summary).

##### 4.3.3.1 Stoffenmanager®

First published by Marquart et al. (50), Stoffenmanager® is considered a Tier 1/Tier 2 hybrid (44) or Tier 1.5 modifying factors tool (26) capable of both inhalation and dermal exposure assessment (see Supplementary Table S10 for tool summary and Supplementary Table S11 for example output report). As of 2020, Stoffenmanager® has over 37,000 users and supported over 310,000 assessments (50). Stoffenmanager® is reported to tend to overestimate low exposures and underestimate high exposures (26) but that using the 90% percentile typically applied sufficient conservatism (34).

During case-study testing Stoffenmanager® was found to be clear and straight forward to use, if a little cumbersome for data entry with a tendency to stutter as the system recalculated (not uncommon in large EHS database/management system tools). A potential risk of user entry error was found for product creation, although paid access to the pure substance database may reduce some of this. Some input options appeared vague, however, once a parameter is selected the system generates real-world examples, allowing the user to calibrate their selection.

The paid subscription provides users the ability to utilize a wider variety of features including the prioritization of exposure risk, access to a pure substances database, stored substance assessments, Safety Data Sheet registry and expert support allowing Stoffenmanager® to act as a more complete chemical management system (51).

#### 4.3.4 Mass-balanced-based models

Mass-balance-based models combine physico-chemical relationships of the substance with the conservation of mass principle to determine exposure risk (50). Fugitive emissions, such as those from “valves, pumps, compressors, flanges, storage tanks, pressure relief valves,” are the primary source of exposure (46). Consequently, fugitive emission source strength is the key parameter for mass-balanced-based models and typically require real world process data. Where this is not available, conservative estimates can be used assuming all losses are emitted to the air. Theoretically, mass-balance-based models provide a greater degree of accuracy due to the high degree of process specificity, however, when practically applied, the assumptions required to compensate for a lack of reliable data, combined with natural process and operator variability can introduce a high degree of uncertainty. The high specificity of these models also reduces their applicability as a general use model (50).

##### 4.3.4.1 SPR-lop hybrid tool

The SPR-Lop Hybrid tool quantifies the hazards from the ‘Source’ chemical(s) and specific operational steps, the potential quantity and properties of hazardous fugitive emissions released through the process ‘Pathway’ and their potential to cause exposure to the ‘Receptor’ while accounting for the ‘Layers of Protection’ (LOP) (46).

The tool outputs three representative values (1) Severity, (2) Probability of leakage, and (3) Probability of exposure. These values are applied to exposure matrices along with an evaluation of the safety culture and an assessment of control effectiveness to output a final risk ranking.

The SRP-LOP Hybrid tool provides a potentially powerful methodology for comprehensive exposure assessment; however, significant process information and expertise would be required to utilize this method properly. Additionally, while the measurements of the strength of the safety culture and management system are important when considering the uncertainty level associated with the results of an exposure assessment (26), the validity of assigning assessments conducted in this way, as a modifier for exposure potential is less certain. There may also be concerns about the accuracy of measuring the safety culture and management system, as proposed, based on input from a single health and safety representative from the organization, due to the potential for bias and uncertainty from differing levels of competence and personal knowledge of processes, operators, supervisors and inter-shift variability. The degree of personal involvement in design and implementation of the management system could also have the potential to introduce bias.

##### 4.3.4.2 The near field-far field (NF-FF) deterministic model

The Near Field-Far Field model considers the exposure area as two zones, the Near Field (NF) is the immediate workstation and breathing zone of the operator and Far Field (FF) is the full work area within which the process is occurring. The NF-FF model calculates the change in concentration of the source in the NF, its migration to the FF and subsequent concentration reduction. This model makes some key assumptions; that source buildup is at a constant rate, that air flow between the NF and FF zones mix well and that the only contaminant extraction route is ventilation into the FF (42).

The NF-FF method can provide an estimate of the level of exposure over both the 8-h and 15-min-time-weighted-averages, but it does not provide any confidence intervals for these measurements (42) and has limited published use cases. For model validity it would be critical to confirm good air mixing and indoor ventilation characteristics between the NF and FF.

##### 4.3.4.3 Modifying-factor, mass-balance combination models

###### 4.3.4.3.1 The advanced reach tool (ART)

First published by Fransman et al. (50) ART is a fully published Tier 2 model, which applies modifying factors to a NF-FF framework. The ART tool utilizes a Bayesian methodology where the mechanistic model can be updated with real-world exposure data for “improved accuracy and precision” (44, 45) (see Supplementary Table S12 for ART model summary).

ART’s quantitative estimates have been calibrated with over three thousand air sampling measurements (26), and it has a strong correlation to air monitoring results at higher concentrations and a greater degree of precision and accuracy compared to Stoffenmanager® (34). This reflects the incorporation of Bayesian techniques in the ART model thus enabling model calibration. Some tests found that ART may tend to overestimate exposure at lower concentrations (26, 52).

The case study testing of ART (see Supplementary Table S13) found its user interface was simple to use and while it has a higher requirement for complex data entry than other tools, the system provides drop down menus and guidance to support selection. ART easily facilitates the combined assessment of a multistep process but unlike other tools the decision of exposure acceptability lies with the user.

#### 4.3.5 Model uncertainty and validity

In general terms, “in deterministic models, the output of the model is fully determined by the parameter values and the initial values, whereas probabilistic (or stochastic) models incorporate randomness in their approach. Consequently, the same set of parameter values and initial conditions will lead to a group of different outputs [refer PreventionWeb (53)]. While the former models use fixed values as input variables the latter are based on probability distributions enabling a greater degree of variability to be incorporated into the model inputs.

All models have a degree of error, Koivisto et al. describes these as errors inherent to the design of system multipliers and errors in the judgment by the group decisions of the expert panel. The other key type of error comes from the user, where the available options do not suitably reflect the real-world scenario, or the user mis-interprets the intent of the system designers and incorrectly applies a modifier (54).

Shandilya et al. raises concerns about the use of ‘dustiness’ in several models including the ART and Stoffenmanager®, concluding that moisture content was not a linear effect, but rather a curve with an inflection point at 4%, and as a result the qualitative measurement used is “too conservative and insufficient” for accurate exposure estimation (45).

While the theoretical models behind Stoffenmanager® and ART have been openly published, to date, not all calibration measurements have been made publicly available (50, 54). Calibrating a model across various processes and workplaces will reduce bias as it enables coverage of a large variety of real-world settings. Sometimes the model calibration may result in errors due to nature of data and methodology used and (54) these concerns were highlighted at the ISES exposure models working group in 2022 but consensus on calibration validity could not be reached (50).

#### 4.3.6 Other useful tools

The following section notes additional tools identified during the literature review that may be suitable for modelers with specific exposure assessment needs.

##### 4.3.6.1 Industrial hygiene exposure scenario tool (IHEST)

Available from the AIHA, this spreadsheet tool provides guidance for basic characterization of a broad range of exposure scenarios. The tool assists the assessor through standardization of data gathering and cataloging important scenario information (31).

##### 4.3.6.2 Control band control measures tables

Groso et al. (55) presented a very simple table format that could be of interest to multi-product facilities handling materials from different control bands.

### 4.4 Exposure model recommendations to the biopharmaceutical industry

**Figure 2:**
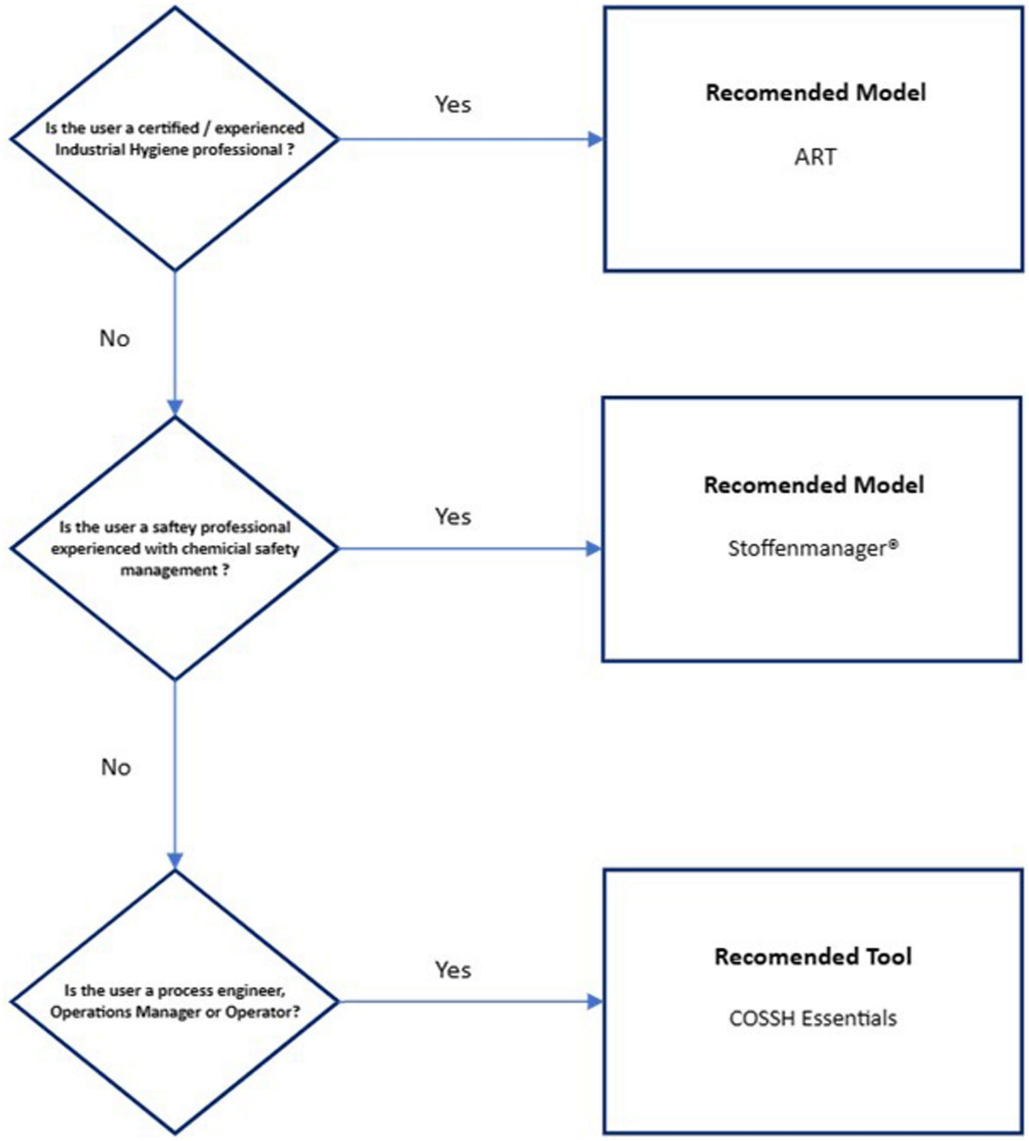
Ranked recommendation for model use in biopharmaceutical industry.

Figure 2 presents a ranked recommendation to the biopharmaceutical industry for model use based on worker job role/presumed level of chemical exposure expertise. ART has been recommended only for certified/experienced industrial hygienists due to the inherent complexity of the model and that the model provides an exposure confidence interval that needs to be interpreted against an appropriate exposure standard. Stoffenmanager® has been recommended for use by Safety professionals experienced in chemical safety management due to the reduced, but still high level of complexity, on the basis of expert support availability. Validation of both models against existing organizational exposure data is strongly recommended, especially exposure modeling of OEB 4 and 5 substances. The COSSH Essentials tool has been recommended for process engineers, operations managers, and operators as it is highly conservative and may provide useful guidance on the application of exposure controls.

### 4.5 Future research needs

While no recent studies were identified that specifically validated any exposure models for use in multiple scenarios associated with biopharmaceutical manufacture it is further recommended that a trial application of ART, Stoffenmanager® and COSSH be undertaken across SEG groups in the biopharmaceutical manufacturing industry. This would provide valuable information that could eventually lead to exposure model accuracy and validity for common API and DP operations including maintenance across that industry.

## 5 Conclusion

A systematic literature search was conducted to identify the most recent and well-published occupational exposure models that could be of value to the biopharmaceutical industry. Seven reported occupational models were shortlisted for critical review, and within these, several key model mechanisms were identified and critically assessed. These included:

- Simple control banding tools apply standardized controls to discrete exposure risk levels. These models are highly conservative but require limited expertise to use.
- Heuristic algorithms (e.g., SDM 2.0) apply standardized rule sets to a limited set of substance property data resulting in a conservative exposure estimate. These models require a greater level of user expertise than simple CB models and have limited application.
- Multiplying factor-based models which reduce the substances and process parameters down to a discrete set of variables with each applying weighting to an equation. Higher tier multiplying factors models, such as Stoffenmanager®, can provide quantitative exposure estimates with a measure of uncertainty.
- Mass-balance based models combine physical and chemical data of the source with an estimate of fugitive emissions from the process and their interaction with workers in the workspace. While these models can have a high degree of accuracy, the large amount of real-world process emissions data potentially makes them redundant as a tool to minimize the need for air sampling.

The only highly investigated Tier 2 inhalation exposure model identified was the ART tool. ART uses multiplying factors applied to a NF-FF framework to provide an estimate of worker exposure with confidence intervals. ART is the most accurate of the multiplying factors models reviewed and allows for the integration of real-work sampling data into the model to facilitate Bayesian analysis.

As occupational exposure models increase in predictive capability they increase in complexity and require a greater level of expertise to correctly use and interpret. Concerns have also been raised about the validity of generating quantitative exposure estimates with uncertainty measures from what is essentially qualitative inputs. This is in addition to concerns that the calibration conducted on these tools using real-world air sampling data has not been made publicly available, may not be appropriate, and could increase the level of error.

There is no fully accepted, accurate and fully validated exposure model available to the biomanufacturing industry. Nor is it possible to conduct air monitoring for every possible occupational exposure scenario that may occur within these workplaces. On the basis that there is no perfect alternative, we propose that ART, when used by a qualified/experienced industrial hygienist, at an appropriate confidence level, is the most suitable currently available exposure model to the biopharmaceutical manufacturing industry.

Stoffenmanager® offers value as an occupational exposure model for use by safety professionals on the basis that its paid model includes expert guidance and support. Both ART and Stoffenmanager® should be validated for accuracy against existing organizational air monitoring data from the specific production environments in which they will be applied.

The COSHH e-tool can provide useful guidance to process engineers, managers and workers on appropriately conservative exposure controls needed to protect workers and the IHSET tool provides a useful way for standardizing the data collection when investigating exposure scenarios.

Future recommended research is therefore to validate the use of ART and Stoffenmanager® for common API and DP manufacturing operations, especially for higher OEB substances with very low permissible exposure levels.

## 6 Limitations

The purpose of this study was not to re-evaluate identified exposure models but to find and apply potentially useful tools to the context of a case study; biopharmaceutical development and manufacturing at ‘light speed.’ Applying identified exposure models to different case studies using professionals with differing qualifications may have different results.
