# Supplementary material for: Occupational hygiene risk assessment at light speed—a study for protecting worker health and safety in the biopharmaceutical industry
Source: Front Public Health. 2025 Jun 25;13:1559588. doi: 10.3389/fpubh.2025.1559588 (PMC12238065; doi:10.3389/fpubh.2025.1559588)

Supplementary Material

Occupational Hygiene Risk Assessment at Light Speed; A Study for Protecting Worker Health and Safety in the Biopharmaceutical Industry

This supplementary material includes 13 supplementary tables.

## S1 Table: Pubmed Search Strategy.

| Occupational Hygiene | Risk Assessment | Therapeutics |
| --- | --- | --- |
| “Occupational Health”[mh] OR  Occupational Health[tiab] OR  Industrial Hygiene[tiab] OR  Industrial Health[tiab] OR  Occupational Safety[tiab] OR  Employee Health[tiab] OR  workplace hazard*[tiab] OR  workplace health[tiab] OR  workplace safety[tiab] OR  employee safety[tiab] OR  "Occupational Exposure"[mh] OR  Occupational Exposure*[tiab] OR  "Occupational Exposure / analysis"[tiab] OR  Occupational Exposure / analysis[tiab] OR  Occupational Exposure / prevention & control*[tiab] OR  Occupational Exposure / standards [tiab] OR  “Safety management”[mh] OR  Safety Management[tiab] OR  Hazard Surveillance Program*[tiab] OR  Hazard Management[tiab] OR  Hazard control[tiab] OR  safe handling[tiab] OR  exposure control[tiab] OR  risk management[tiab] OR  Air Pollutants, Occupational / analysis*[tiab] | "Risk Assessment"[mh] OR  Risk Assessment*[tiab] OR  Risk Analysis[tiab] OR  Risk Analyses[tiab] OR  Exposure assessment[tiab] OR  Qualitative exposure assessment tool[tiab] OR  Exposure assessment model[tiab] OR  "Algorithm*"[mh] OR  Algorithm*[tiab] OR  "Judgment"[mh] OR  Judgment [tiab] OR  "Checklist"[mh] OR  Checklist[tiab] OR  "Decision making"[mh] OR  Decision making[tiab] | "Biological Products"[mh] OR  Biological product*[tiab] OR Biopharmaceutical*[tiab] OR  Biologic*[tiab] OR  Biological drug*[tiab] OR  Biological medicine[tiab] OR  Biological Pharmaceutical*[tiab] OR  biopharmaceutical product*[tiab] OR  biopharmaceutical drug*[tiab] OR  “pharmaceutical preparations” [mh] OR  pharmaceutical preparation*[tiab] |
| 65,478 results | 1,457,412 results | 2,434,455 results |
| #1 AND #2 AND #3 = 1,002 results | | |
| #1 AND #2 AND #3 (Filters applied: last 10 yrs, English only, Humans only) = 346 results | | |
| Results moved to Endnote | | |

## S2 Table: Web of Science Search Strategy

| Occupational Hygiene | Risk Assessment | Therapeutics |
| --- | --- | --- |
| (“Occupational Health” OR  "Industrial Hygiene" OR  "Industrial Health" OR  "Occupational Safety" OR  "Employee Health" OR  "workplace hazard*" OR  "workplace health" OR  "workplace safety" OR  "employee safety" OR  "Occupational Exposure" OR  "Occupational Exposure*" OR  "Occupational Exposure analysis" OR  "Occupational Exposure prevention & control*" OR  "Occupational Exposure standard*" OR  “Safety management” OR  "Hazard Surveillance Program*" OR  "Hazard Management" OR  "Hazard control" OR  "safe handling" OR  "exposure control" OR  "risk management" OR  "Air Pollutants, Occupational analys*") | ("Risk Assessment" OR  "Risk Assessment*" OR  "Risk Analysis" OR  "Risk Analyses" OR  "Exposure assessment" OR  "Qualitative exposure assessment tool" OR  "Exposure assessment model" OR  "Algorithm*" OR  "Algorithm*" OR  "Judgment" OR  "Checklist" OR  "Decision making") | ("Biological Products" OR  "Biological product*" OR  "Biopharmaceutical" OR  "Biologic*" OR  "Biological drug*" OR  "Biological medicine" OR  "Biological Pharmaceutical*" OR  "biopharmaceutical product*" OR  "biopharmaceutical drug*" OR  "pharmaceutical preparations" OR  "pharmaceutical preparation*") |
| 64,887 results | 3,615,669 results | 2,441,427 results |
| #1 AND #2 AND #3 = 551 results | | |
| #1 AND #2 (Filters applied : Date 2013 – 2023; Research area – Public environmental Occupational Health; English only, English only; = 78 results | | |
| Results moved to Endnote | | |

## S3 Table: Scopus search Strategy

| Occupational Hygiene | Risk Assessment | Therapeutics |
| --- | --- | --- |
| “Occupational Health” OR  "Industrial Hygiene" OR  "Industrial Health" OR  "Occupational Safety" OR  "Employee Health" OR  "workplace hazard*" OR  "workplace health" OR  "workplace safety" OR  "employee safety" OR  "Occupational Exposure" OR  "Occupational Exposure*" OR  "Occupational Exposure / analysis" OR  "Occupational Exposure / analysis" OR  "Occupational Exposure / prevention & control*" OR  "Occupational Exposure / standard*" OR  “Safety management” OR  "Hazard Surveillance Program*" OR  "Hazard Management" OR  "Hazard control" OR  "safe handling" OR  "exposure control" OR  "risk management" OR  "Air Pollutants, Occupational / analys*" | "Risk Assessment" OR  "Risk Assessment*" OR  "Risk Analysis" OR  "Risk Analyses" OR  "Exposure assessment" OR  "Qualitative exposure assessment tool" OR  "Exposure assessment model" OR  "Algorithm*" OR  "Algorithm*" OR  "Judgment" OR  "Checklist" OR  "Decision making" | "Biological Products" OR  "Biological product*" OR  "Biopharmaceutical" OR  "Biologic*" OR  "Biological drug*" OR  "Biological medicine" OR  "Biological Pharmaceutical*" OR  "biopharmaceutical product*" OR  "biopharmaceutical drug*" OR  "pharmaceutical preparations" OR  "pharmaceutical preparation*" |
| 1,223,709 results | 12,145,946 results | 14,550,240 results |
| #1 AND #2 AND #3 = 95,354 results | | |
| #1 AND #2 AND #3  (Limit to:  Document type - Article, Review, Conference paper, and book chapter; Subject Area – Environmental sciences, Decision sciences, chemistry, Health Professions, Multidisciplinary;  Publication – International Journal Of Environmental Research And Public Health, Environmental Health Perspectives, Plos One, Toxicology And Industrial Health, Industrial Health, Reviews On Environmental Health, Journal Of Toxicology And Environmental Health Part A, Archives Of Environmental Health, Journal Of Exposure Analysis And Environmental Epidemiology, Human And Ecological Risk Assessment, Archives Of Environmental And Occupational Health, International Journal Of Environmental Health Research, Proceedings Of The National Academy Of Sciences Of The United States Of America, Journal Of Toxicology And Environmental Health, Toxicology And Environmental Health Sciences, Current Environmental Health Reports, Journal Of Environmental And Public Health, Process Safety And Environmental Protection, Science, Noise And Health, Journal Of Environmental Health  Keywords – Human, Humans, Risk Assessment;  Date Range – 2013 to 2024) = 3,482 results | | |
| Results moved to Endnote | | |

## S4 Table: Full text articles excluded with reasons, presented in alphabetical order by first author.

| **Reference** | **Exclusion justification** |
| --- | --- |
| Askari, A., Poursadeqiyan, M., Sahl Abadi, A. S., Mahdinasab, L., & Farhadi, A. R. (2023). Semi-quantitative risk assessment for workers exposed to occupational harmful agents in an oilfield in Iran. Work, 76(1), 147-157. | Full text article not available through university subscription |
| Graham, J. C., Hillegass, J., & Schulze, G. (2020). Considerations for setting occupational exposure limits for novel pharmaceutical modalities. Regul Toxicol Pharmacol, 118, 104813. | Wrong subject of study  Paper’s focus is toxicology, methodologies for determination of appropriate occupational exposure limits and not modelling / qualitative assessment of exposure against said limits |
| Guha, N., Guyton, K. Z., Loomis, D., & Barupal, D. K. (2016). Prioritizing chemicals for risk assessment using chemoinformatics: Examples from the IARC monographs on pesticides. Environmental Health Perspectives, 124(12), 1823-1829. | Wrong subject of study  Paper’s focus is toxicology, determination likely oncolytic properties of substances by analysing their monograph |
| Gul, M., Ak, M. F., & Guneri, A. F. (2017). Occupational health and safety risk assessment in hospitals: A case study using two-stage fuzzy multi-criteria approach. Human and Ecological Risk Assessment, 23(2), 187-202. | Wrong subject of study  The report focus is a general health and safety risk assessment methodology and not specific to occupational hygiene exposure assessment. |
| Lynch, H. N., Allen, L. H., Hamaji, C. M., & Maier, A. (2023). Strategies for refinement of occupational inhalation exposure evaluation in the EPA TSCA risk evaluation process. Toxicology and Industrial Health, 39(3), 169-182. | Wrong type of study  Modelling was undertaken using aggregated air sampling data. |
| Miller, M. F., Goodson, W. H., Manjili, M. H., Kleinstreuer, N., Bisson, W. H., & Lowe, L. (2017). Low-dose mixture hypothesis of carcinogenesis workshop: Scientific underpinnings and research recommendations. Environmental Health Perspectives, 125(2), 163-169. | Wrong population  The study was focused on general population exposure rather than exposure specific to occupational settings |
| Niemeier, R. T., Williams, P. R. D., Rossner, A., Clougherty, J. E., & Rice, G. E. (2020). A cumulative risk perspective for occupational health and safety (OHS) professionals. International Journal of Environmental Research and Public Health, 17(17), 1-19. | Wrong population  Assessment type is not specific to occupational chemical exposure. |
| Peters, S., Vienneau, D., Sampri, A., Turner, M. C., Castaño-Vinyals, G., Bugge, M., & Vermeulen, R. (2022). Occupational Exposure Assessment Tools in Europe: A Comprehensive Inventory Overview. Annals of Work Exposures and Health, 66(5), 671-686. | Wrong subject of study  Study reports comprehensive list of Job Exposure matrices resulting from exposure assessments not exposure assessment methodologies. |
| Samantra, C., Datta, S., & Mahapatra, S. S. (2017). Analysis of occupational health hazards and associated risks in fuzzy environment: a case research in an Indian underground coal mine. Int J Inj Contr Saf Promot, 24(3), 311-327. | Wrong subject of study  The report focus is a general health and safety risk assessment methodology and not specific to occupational hygiene exposure assessment. |
| Solomon, K. R., Wilks, M. F., Bachman, A., Boobis, A., Moretto, A., Pastoor, T. P., Phillips, R., & Embry, M. R. (2016). Problem formulation for risk assessment of combined exposures to chemicals and other stressors in humans. Crit Rev Toxicol, 46(10), 835-844. | Wrong population  Assessment type is not specific to occupational chemical exposure. |
| Sousa, M., Arezes, P., & Silva, F. (2021). Occupational exposure to ultrafine particles in metal additive manufacturing: A qualitative and quantitative risk assessment. International Journal of Environmental Research and Public Health, 18(18). | Wrong subject of study  Assessment methodology is highly specific for metal manufacturing, not applicable to biopharma setting. |
| Sussman, R. G., Schatz, A. R., Kimmel, T. A., Ader, A., Naumann, B. D., & Weideman, P. A. (2016). Identifying and assessing highly hazardous drugs within quality risk management programs. Regul Toxicol Pharmacol, 79, S11-18. | Wrong subject of study  Study relates to GMP manufacturing quality risk assessment as opposed to occupational health exposure assessment. |
| Wheeler, D. C., Rustom, S., Carli, M., Metayer, C., Whitehead, T. P., & Ward, M. H. (2021). Assessment of grouped weighted quantile sum regression for modeling chemical mixtures and cancer risk. International Journal of Environmental Research and Public Health, 18(2), 1-20. | Wrong population  Assessment type is not specific to occupational chemical exposure. |
| Wignall, J. A., Muratov, E., Sedykh, A., Guyton, K. Z., Tropsha, A., Rusyn, I., & Chiu, W. A. (2018). Conditional toxicity value (CTV) predictor: An in silico approach for generating quantitative risk estimates for chemicals. Environmental Health Perspectives, 126(5). | Wrong subject of study  Paper’s focus is toxicology, methodologies for determination of appropriate occupational exposure limits and not modelling / qualitative assessment of exposure against said limits |
| Yang, J. H., Kim, H. S., Koo, B. K., Lee, C. M., Jung, J. H., & Seo, Y. R. (2018). Considerations of Human Health Risk Assessment in Chemical Accident: Suggestions from a Toxicogenomic Approach. Toxicology and Environmental Health Sciences, 10(2), 79-89. | Wrong population  Assessment type is not specific to occupational chemical exposure. |
| Zhu, J., Liu, Z., Cao, Z., Han, X., Hao, L., & Wei, H. (2022). Development of a general inherent safety assessment tool at early design stage of chemical process. Process Safety and Environmental Protection, 167, 356-367. | Wrong subject of study  Paper focus is generalized to process safety management risk assessment rather than specific to occupational exposure assessment. |

## S5 Table: Full text articles included into synthesis / critical review presented in alphabetical order by first author.

| Alhamdani, Y. A., Hassim, M. H., Shaik, S. M., & Jalil, A. A. (2018). Hybrid tool for occupational health risk assessment and fugitive emissions control in chemical processes based on the source, path and receptor concept. Process Safety and Environmental Protection, 118, 348-360. |
| --- |
| Arnold, S., Stenzel, M., & Ramachandran, G. (2015). Approaches to Improving Professional Judgment Accuracy. In A Strategy for Assessing and Managing Occupational Exposures (pp. 80-94). (Reprinted from 4) |
| Arnold, S. F., Stenzel, M., Drolet, D., & Ramachandran, G. (2016). Using checklists and algorithms to improve qualitative exposure judgment accuracy. Journal of Occupational and Environmental Hygiene, 13(3), 159-168. |
| Bekker, C., Voogd, E., Fransman, W., & Vermeulen, R. (2016). The Validity and Applicability of Using a Generic Exposure Assessment Model for Occupational Exposure to Nano-Objects and Their Aggregates and Agglomerates. Ann Occup Hyg, 60(9), 1039-1048. |
| Cherrie, J. W., Fransman, W., Heussen, G. A. H., Koppisch, D., & Jensen, K. A. (2020). Exposure models for reach and occupational safety and health regulations. International Journal of Environmental Research and Public Health, 17(2). |
| Gridelet, L., Delbecq, P., Hervé, L., Boissolle, P., Fleury, D., Kowal, S., & Fayet, G. (2015). Proposal of a new risk assessment method for the handling of powders and nanomaterials. Ind Health, 53(1), 56-68. |
| Groso, A., Petri-Fink, A., Rothen-Rutishauser, B., Hofmann, H., & Meyer, T. (2016). Engineered nanomaterials: toward effective safety management in research laboratories. J Nanobiotechnology, 14, 21. |
| Hofstetter, E., Spencer, J. W., Hiteshew, K., Coutu, M., & Nealley, M. (2013). Evaluation of recommended REACH exposure modeling tools and near-field, far-field model in assessing occupational exposure to toluene from spray paint. Ann Occup Hyg, 57(2), 210-220. |
| HSE, U. (2017). Controlling Exposure to Chemicals–A Simple Control Banding Approach. In. UK: COSHH e-tool. |
| Huizen, D. (2023). Exposure Assessments: What are the Steps to Properly Determine Workplace Exposures Before Sampling? Michigan Safety Conference, Grand Rapids, Michigan. |
| Kimbrough, L. J., Oestenstad, R. K., & Beasley, T. M. (2020). Evaluation of the exposure prediction component of Control of Substances Hazardous to Health Essentials. J Occup Environ Hyg, 17(2), 97-108. |
| Koivisto, A. J., Jayjock, M., Hämeri, K. J., Kulmala, M., Van Sprang, P., Yu, M., Boor, B. E., Hussein, T., Koponen, I. K., Löndahl, J., Morawska, L., Little, J. C., & Arnold, S. (2021). Evaluating the Theoretical Background of STOFFENMANAGER® and the Advanced REACH Tool. Annals of Work Exposures and Health, 66(4), 520-536. |
| Sailabaht, A., Wang, F., & Cherrie, J. (2018). Extension of the advanced REACH tool (ART) to include welding fume exposure. International Journal of Environmental Research and Public Health, 15(10). |
| Schlüter, U., Arnold, S., Borghi, F., Cherrie, J., Fransman, W., Heussen, H., Jayjock, M., Jensen, K. A., Koivisto, J., Koppisch, D., Meyer, J., Spinazzè, A., Tanarro, C., Verpaele, S., & von Goetz, N. (2022). Theoretical Background of Occupational-Exposure Models—Report of an Expert Workshop of the ISES Europe Working Group “Exposure Models”. International Journal of Environmental Research and Public Health, 19(3). |
| Shandilya, N., Kuijpers, E., Tuinman, I., & Fransman, W. (2019). Powder Intrinsic Properties as Dustiness Predictor for an Efficient Exposure Assessment? Ann Work Expo Health, 63(9), 1029-1045. |
| Spinazzè, A., Borghi, F., Campagnolo, D., Rovelli, S., Keller, M., Fanti, G., Cattaneo, A., & Cavallo, D. M. (2019). How to Obtain a Reliable Estimate of Occupational Exposure? Review and Discussion of Models' Reliability. International Journal of Environmental Research and Public Health, 16(15), 2764-. |

**S6 Table:** Structured Deterministic Model (SDM) 2.0 summary.

| **Structured Deterministic Model (SDM) 2.0** | | |
| --- | --- | --- |
| **Model Type** | Heuristic |  |
| **Endorsing body** | AIHA |  |
| **Model Tier** | Tier 1 |  |
| **Modelling Capability** | Gases, volatile and semi-volatile liquids | Checklist 1 |
|  | Aerosols, particulates and Fibres | Checklist 2 |
| **Inputs for checklist 1** | Scenario name | Free text |
|  | Date |  |
|  | Temp | Quantitative |
|  | Scenario number | Discrete options |
|  | Substance(s) (from data base or user defined) | Discrete options |
|  | Vapour pressure (from data base or user defined) | Quantitative |
|  | Select OEL (from data base of user defined) | Quantitative |
|  | Repeat steps for additional mixture components |  |
| **Output for checklist 1** | Predicted exposure concentration under different control conditions |  |
|  | Health hazard rating using different exposure controls | A-E |
| **Exposure estimate** | Yes |  |
| **Uncertainty measures** | 95th percentile |  |
| **Inputs for checklist 2** | Exposure range | Discrete options |
|  | Observed controls | Discrete options |
| **Output for checklist 2** | Predicted exposure category | Qualitative |
| **Exposure estimate** | No |  |
| **Uncertainty measures** | No |  |

**S7 Table 4:** COSHH e-tool summary

| **COSHH e-tool** | | |
| --- | --- | --- |
| **Model Type** | Control Banding control guidance tool |  |
| **Endorsing body** | HSE |  |
| **Model Tier** | NA |  |
| **Modelling Capability** | Solids or liquids |  |
| **Inputs** | Process name and type | Free text |
|  | H- codes | Tick box |
|  | For Liquids - Vapour pressure and temperature | Quantitative |
|  | Dustiness - for powders | Discrete options |
|  | Quantity used | Discrete options |
|  | Task frequency | Quantitative |
|  | Task Duration | Quantitative |
| **Output** | Assessment Summary |  |
|  | Qualitative chemical hazard rating | A-E |
|  | Links to applicable exposure control guidance |  |
| **Exposure estimate** | No |  |
| **Uncertainty measures** | No |  |

**S8 Table:** Output of COSSH Essentials e-tool for the preparation of an HCl stock solution from concentrated acid.


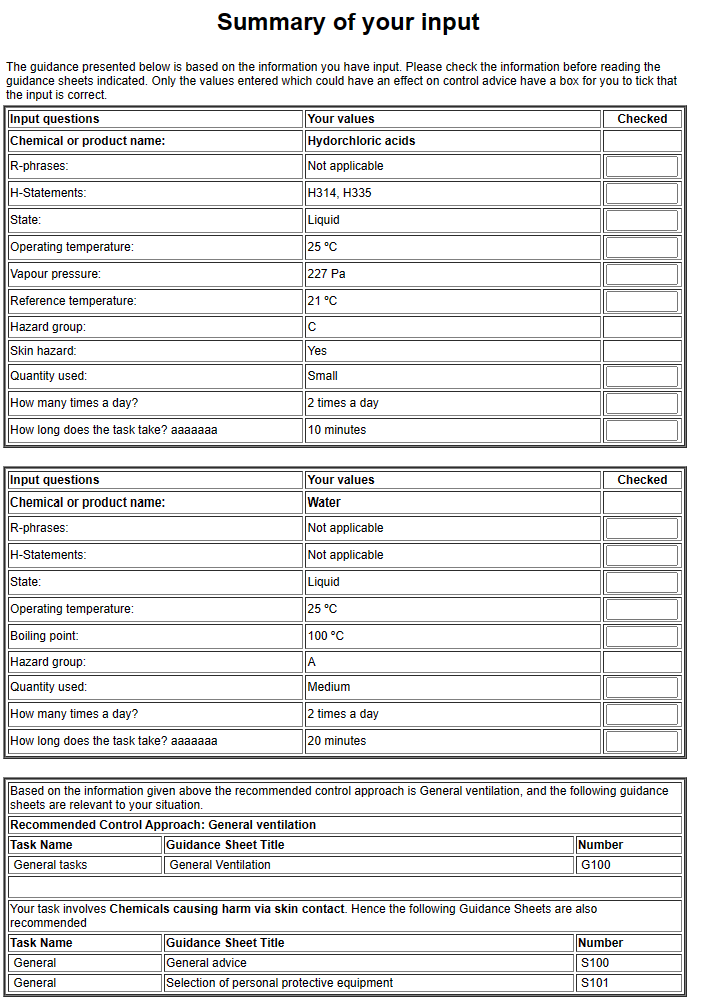


**S9 Table:** Gridelet et al., (2015) Multiplying Factors Model Summary.

| **Gridelet et al., (2015) Multiplying Factors Model** | | |
| --- | --- | --- |
| **Model Type** | Multiplying factors Control banding tool | paper based |
| **Endorsing body** | Not identified |  |
| **Model Tier** | Not identified |  |
| **Modelling Capability** | Powders and nano materials |  |
| **Inputs** | Occupational Hazard Band (OHB) | Discrete options |
|  | Hermeticity (H) | Discrete options |
|  | Dustiness (S) | Discrete options |
|  | Emission potential (E) | Discrete options |
|  | Level of containment (C) | Discrete options |
|  | Quantity used (Q) | Discrete options |
|  | Task frequency (F) | Discrete options |
|  | Task Duration (D) | Discrete options |
| **Output** | Exposure indices (Iex) | Quantitative |
|  | Uses risk matrix to compare Iex vs OHB | Qualitative |
|  | Rating of exposure acceptability | Qualitative |
| **Exposure estimate** | No |  |
| **Uncertainty measures** | No |  |

**S10 Table:** Stoffenmanager® Model Use Summary.

| **Stoffenmanager® 8** | | |
| --- | --- | --- |
| **Model Type** | Multiplying factors control banding tool | Web based |
| **Endorsing body** | ECHA |  |
| **Model Tier** | 1.5 (tier 1/tier 2 hybrid) |  |
| **Modelling Capability** | Inhalation and dermal exposure to vapours, low volatility liquid aerosols, and dust. |  |
| **Substance Inputs** | Product name | Qualitative |
|  | Supplier | Qualitative |
|  | Physical properties (gas, solid, fume* etc) | Qualitative |
|  | Vapour pressure / temperature | Quantitative |
|  | SDS | file upload |
|  | SDS expiry date |  |
|  | Exposure controls (expert judgement needed) | Discrete options |
|  | H codes | Discrete options |
|  | P codes | Discrete options |
|  | GHS symbols | Discrete options |
|  | Signal word | Discrete options |
|  | Component substance | Discrete options |
|  | Component concentration | Quantitative |
|  | Applicable OEL | Quantitative |
| **Inhalation Risk assessment inputs** | Name |  |
|  | Location |  |
|  | Job title |  |
|  | Product (from product created above) |  |
|  | Dilution percentage (if applicable) | Quantitative |
|  | Process name |  |
|  | Type of task | Discrete options |
|  | Task Duration | Quantitative |
|  | Task Frequency | Discrete options |
|  | Worker breathing zone (less than 1m) | yes / no |
|  | More than 1 worker | yes / no |
|  | Respiratory protection (Select form large list) | large list |
|  | Work area name |  |
|  | Work area volume | Discrete options |
|  | Work area ventilation |  |
|  | Housekeeping | yes / no |
|  | Monthly inspections and maintenance | yes / no |
|  | Local exhaust ventilation | Discrete options |
| **Output** | Task exposure estimate | Quantitative |
|  | 8 hr shift exposure estimate | Quantitative |
|  | Risk Characterization ratio | Quantitative |
| **Exposure estimate** | Yes |  |
| **Uncertainty measures** | Yes - exposure distribution chart and percentiles |  |

*Not all selectable options can be assessed by the system (i.e. wielding fume, grinding etc.).

**S11 Table:** Output generated using the Stoffenmanager® tool for a model HCl stock solution preparation task.


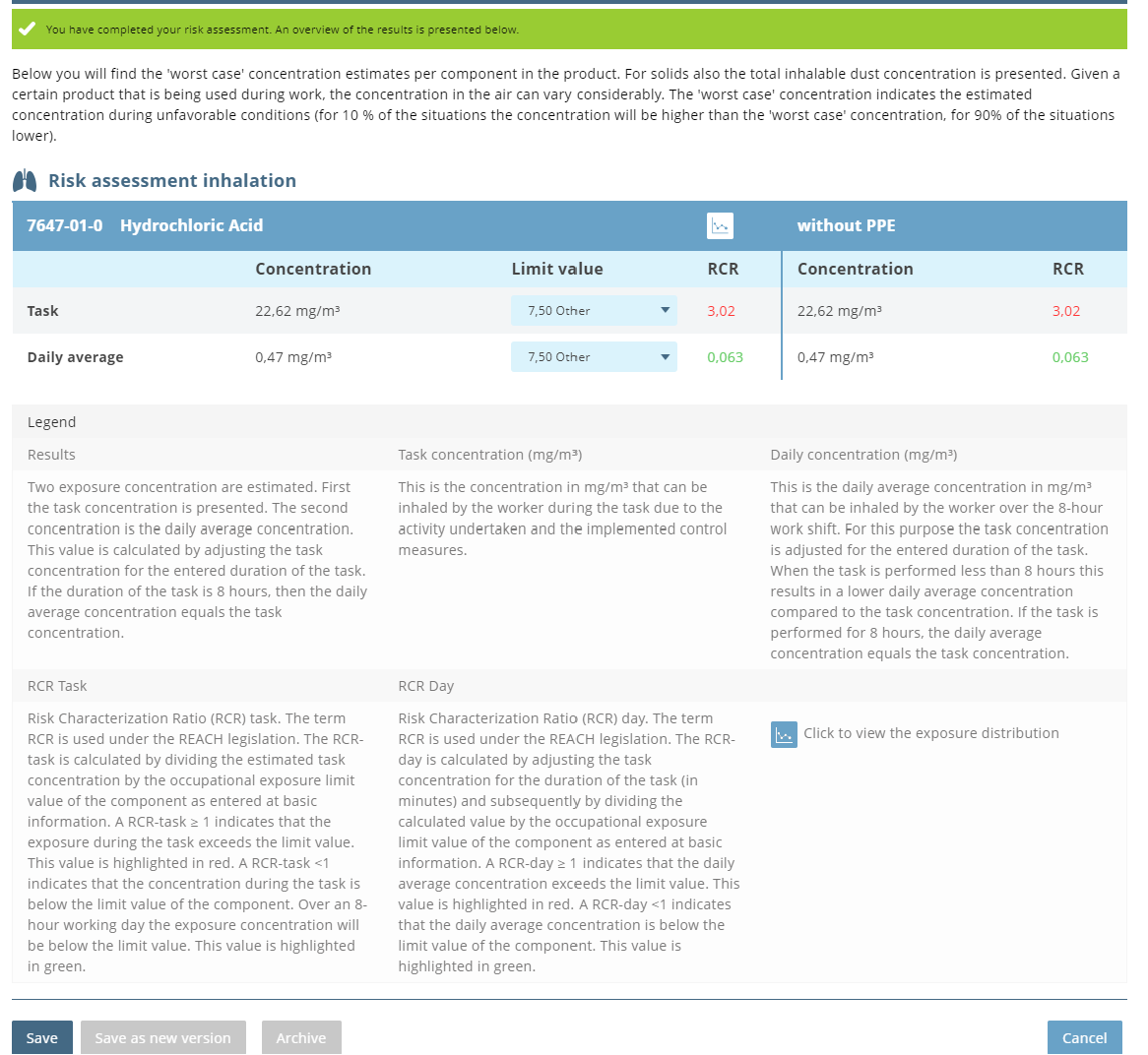


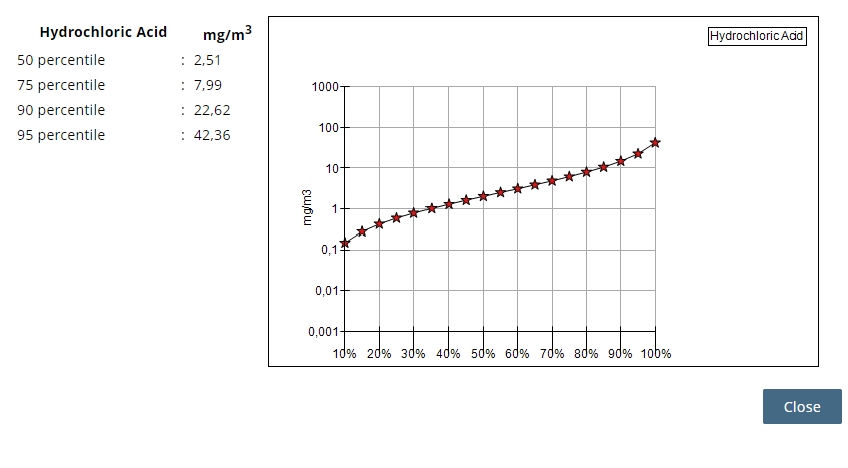


**S12 Table:** ART Model Use Summary.

| **Advanced Reach Tool (ART)** | | |
| --- | --- | --- |
| **Model Type** | Multiplying factors applied to 2 zone NF-FF mass balance framework | Web based |
| **Endorsing body** | ECHA |  |
| **Model Tier** | Tier 2 |  |
| **Modelling Capability** | Inhalation exposure to vapours, dusts and mists. |  |
| **Scenario Identifier** | Name | Free text |
|  | Description | Free text |
| **Substance name** | Substance name |  |
|  | CAS number (if known) |  |
| **Activity configuration (Step 1)** | Step Description | Free text |
|  | Step Duration | Quantitative |
|  | Product type (i.e. powder, liquid, slurry) | Discrete options |
| **For solids** | Wood or Stone |  |
| **For liquids** | Temperature | Quant or Qual |
|  | Vapour pressure | Quantitative |
| **For powders** | Dustiness (for powders) | Quant or Qual |
|  | Moisture content | Discrete options |
| **For paste/ slurry** | Contamination with powdered material | Yes / No |
|  | weight or mole fraction | Quant or Qual |
| **Emission proximity** | Worker breathing zone (less than 1m) | Yes / No |
| **Near field - Emission** | Activity class (i.e. transfer, contaminated object etc) | Discrete options |
|  | Activity subclass (if applicable) | Discrete options |
|  | Activity emission descriptor (ie transfer rate) | Discrete options |
|  | Process containment | Open or closed |
|  | Localised emission controls | Discrete options |
|  | Fugitive emission potential | Yes / No |
|  | Effective housekeeping | Yes / No |
| **Near Field - Dispersion** | Work area (i.e. indoors, outdoors, laminar flow) | Discrete options |
|  | Work area subclass description (i.e. room size) | Discrete options |
|  | Ventilation (room changes per hr) | Discrete options |
| **Near field-secondary emission** | Secondary emission source presence | Yes / No |
|  | (if yes, 2ndary source is processed as above | Discrete options |
| **Additional steps** | Above process repeats for each additional step | Discrete options |
| **Exposure prediction** | Exposure type (Full shift or long term) | Discrete options |
|  | Percentile (50, 75, 90, 95, 99) | Discrete options |
|  | Confidence Interval 80, 90 or 95 | Discrete options |
| **Exposure estimate** | Yes |  |
| **Uncertainty measures** | Yes (Confidence interval) |  |

**S13 Table:** Output generated using ART for a model tank charging exposure assessment.


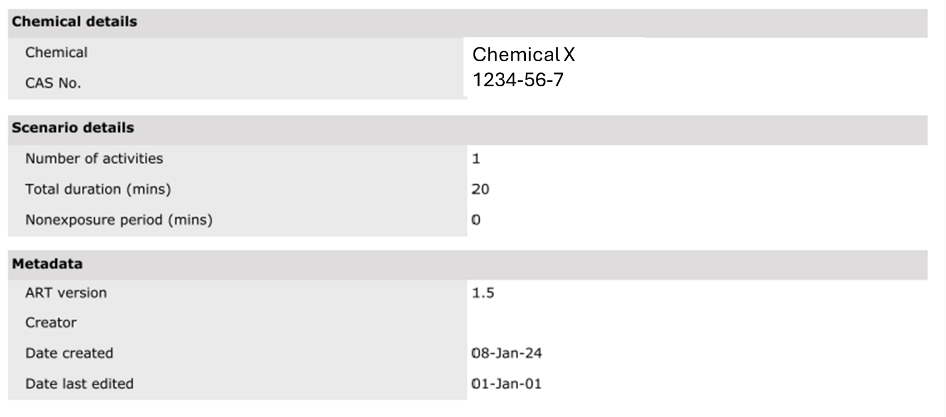


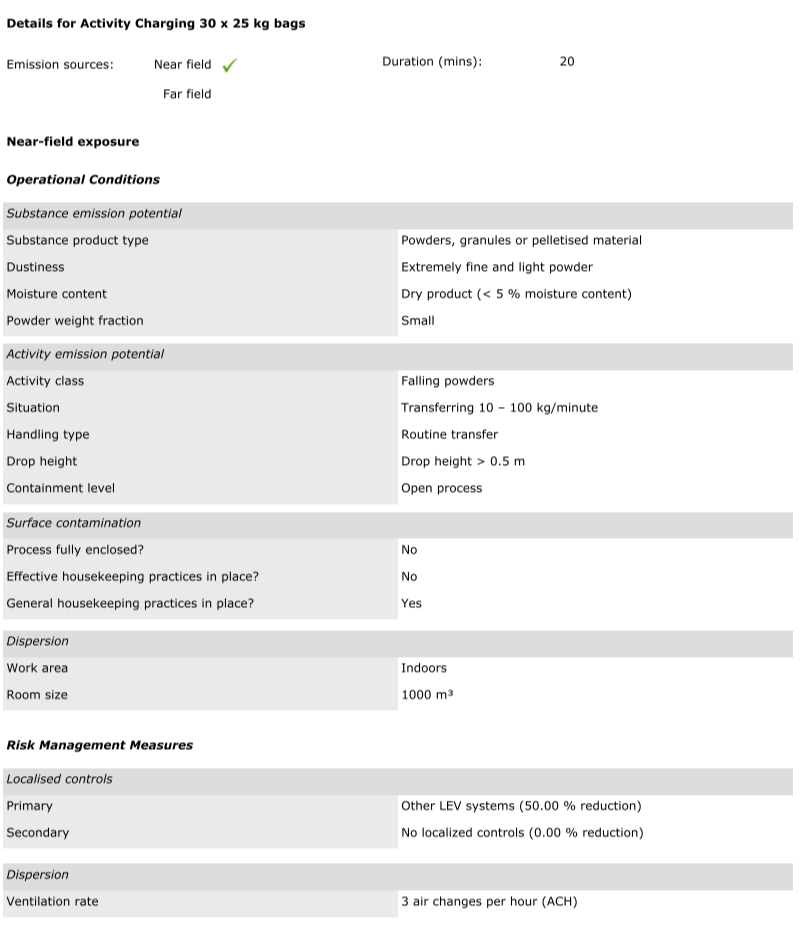


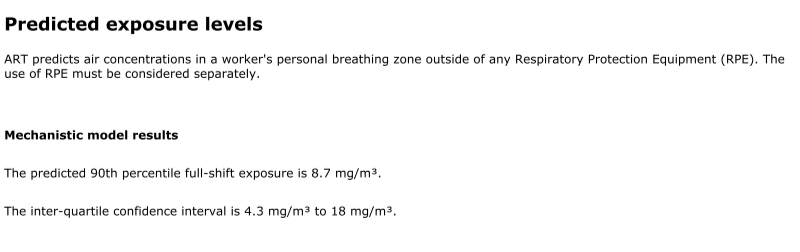

Supplement: Supplementary file 1 [file Table_1.docx]
